# 

**Published:** 1857-11

**Authors:** 


### CONTENTS.

ORIGINAL COMMUNICATIONS— PAGE.
Case of Empyema of the Left Plural Cavity treated by Injection of Iodine. By Daniel Brainard, M.D., ........ 487
Twenty Propositions on Milk-Sickness. By J. W. Crooks, M.D., ........ 489
Veratrum Viride as a Poison and as a Remedial Agent. By J. Stayton Pashley ........ 493
Report of an Amputation in Paris. By John C. Morfit, ........ 494
Milk-Sickness. By John C. Beck, M.D., ........ 496
Translations for the Journal. By Dr. Byford.
Ovarian Cysts, ........ 497
Treatment of the Mamma during Childbed. By Dr. Durr, ........ 499
SELECTIONS—
Sketches in Midwifery Practice. By Walter Channing, M.D., ........ 502
Treatment of Enlarged Bursæ by Caustics. By Jas. B. Prowse, ........ 506
Dr. Theophilus Thompson on the Application of the Microscope to the Diagnosis of Pulmonary Consumption, ........ 507
On the Use of Aconite as a Therapeutic Agent. By Edward B. Stevens, 510
Height of the Pleura above the Clavicle, ........ 512
On the Effect produced on the Circulation by the long-continued Action of Cold Water Externally. By Dr. H. Bence Jones and W. Howship Dickinson, Esq., ........ 512
Photophobia and Blepharospasm relieved by Chloroform, ........ 513
On Bloodletting in Pneumonia. By Prof. Wunderlich, ........ 514
Electricity in the Suppression of the Lacteal Secretion, ........ 516
Sudden Death after Parturition, with Air in the Veins. By George May, Jr., Esq., ........ 516
BOOK NOTICES—
Fisk Fund Prize Essays.
The Effects of Climate on Tubercular Disease. By Edwin Lee, ........ 519
The Influence of Pregnancy on the Development of Tubercles. By Edward Warren, ........ 519
Fourth Report to the General Assembly of Rhode Island, ........ 521
Puerperal Fever—its Causes and Modes of Propagation. By Joseph Smith, M.D., ........ 521
Compound Dislocation of the Long Bones considered with especial reference to the value of resection. By Frank Hastings Hamilton, ........ 523
EDITORIAL—
Personal, ........ 524
Body Snatching—Resurrectionists—The Remedy, ........ 525
Proceedings of the Union Medical Association, ........ 528
ADVERTISEMENTS ........ 531

